# Chronic Insomnia and Stroke Risk—A Real Bidirectional Issue

**DOI:** 10.3390/life15101602

**Published:** 2025-10-14

**Authors:** Brindusa Ilinca Mitoiu, Maria Delia Alexe, Gavril Lucian Gheorghievici, Roxana Nartea

**Affiliations:** 1 Faculty of Medicine, Carol Davila University of Medicine and Pharmacy, 050474 Bucharest, Romania; brindusa.mitoiu@umfcd.ro (B.I.M.); roxana.nartea@umfcd.ro (R.N.); 2 “Prof. Dr. Agrippa Ionescu” Emergency Clinical Hospital, 011356 Bucharest, Romania; 3 National Institute of Rehabilitation Physical Medicine and Balneoclimatology, 3rd Clinic, 030167 Bucharest, Romania

**Keywords:** insomnia, stroke, obesity, arterial hypertension, diabetes, physical inactivity

## Abstract

Chronic insomnia is a prevalent and disabling sleep disorder with growing evidence linking it to cardiovascular and cerebrovascular morbidity. Stroke, a leading cause of mortality and a long-term disability worldwide, may be influenced by sleep disturbances through multiple physiological mechanisms. While traditional risk factors such as hypertension, atrial fibrillation, diabetes, obesity, smoking, and sedentary lifestyle remain dominant drivers of stroke burden, accumulating evidence suggests that sleep disturbances, particularly chronic insomnia, may act as both independent risk factors for incident stroke and as outcomes of cerebrovascular injury. Chronic insomnia, affecting approximately 10% of the global population, is characterized by persistent difficulties with sleep initiation, maintenance, or quality, accompanied by daytime dysfunction. Beyond its impact on quality of life and mental health, insomnia has been linked to cardiometabolic dysregulation, inflammation, and vascular dysfunction. Importantly, sleep disturbances after stroke can impair recovery and functional outcomes, underscoring a bidirectional relationship between stroke and sleep. Several recent reviews have examined the connection between insomnia and stroke. Our review differs by focusing specifically on (1) the stroke-specific epidemiological evidence for chronic insomnia as a risk factor, (2) the bidirectional interplay between insomnia and post-stroke sleep disturbances, and (3) the role of emerging technologies in monitoring and prognosis. By addressing these gaps, we aim to refine the current understanding and highlight priorities for future research and clinical translation.

## 1. Introduction

Stroke is one of the leading causes of mortality worldwide, and it is estimated that 1 in 4 adults will have a stroke throughout their lifetime [1,2,3,4,5,6]. The burden of stroke (one of the most prevalent causes of mortality and disability worldwide) is mainly due to a variety of modifiable risk factors, including hypertension, atrial fibrillation, obesity, disorders of glucose metabolism, smoking, and physical inactivity [7,8,9,10]. Apart from these traditional risk factors, sleep disturbances are becoming more recognized as independent risk factors for stroke and as potential outcomes of stroke. Additionally, the presence or treatment of sleep issues may affect stroke recovery and functional outcomes. Thus, it is important to explore the intricate reciprocal relationship between sleep disturbances and stroke in order to create innovative/new approaches to stroke therapy and prevention.

About 10% of people worldwide suffer from chronic insomnia, one of the most prevalent sleep disorders [4,5,11,12]. It represents a significant public health issue by influencing the quality of life and contributing to the onset or worsening of other comorbid conditions [2,13,14].

Chronic insomnia disorder is characterized by challenges/difficulties in initiating or maintaining sleep, alongside the experience of non-restorative sleep and daytime dysfunction. This disorder can include fatigue, distressed mood, irritability, reducing tolerance to pain and cognitive impairment [15,16]. According to accepted clinical criteria, these symptoms must occur at least three times a week and keep going for at least three months. Since the diagnosis of chronic insomnia disorder primarily relies on clinical assessment, the effects of this sleep disorder need to be examined [16]. Standardized tools like the Pittsburgh Sleep Quality Index (PSQI) and the Epworth Sleepiness Scale (ESS) can enhance this evaluation [17]. The PSQI evaluates sleep quality over the past month, aiming to identify dysfunctional patterns and the severity of the issue currently; a score of 5 or higher suggests that the individual’s sleep quality is deteriorating. The ESS assesses the likelihood of the patient dozing off during daily activities; to be considered as experiencing excessive daytime sleepiness, the patient must achieve a global score of 10 or more [1]. Because diagnosis is based on clinical interviews and patient report, objective measurement (actigraphy, polysomnography) is underutilized in epidemiological studies.

Sleep disturbances have increasingly been recognised as both possible risk factors for stroke and sequelae of stroke, with implications in rehabilitation programs, functional outcomes, and recurrence. The relationship is bidirectional, and its mechanisms remain incompletely elucidated. While prior reviews [7] have summarized the association between insomnia, sleep quality, and cerebrovascular disease, this review aims to extend that literature in several ways:

- By focusing on recent prospective and longitudinal studies from the last decade that address insomnia or chronic sleep disorders as exposure and incident stroke as outcome;
- By emphasizing stroke (not just general cardiovascular disease) and making explicit mechanistic links specific to cerebrovascular pathology;
- By integrating evidence on post-stroke sleep disturbances (epidemiology and functional consequences), which is often omitted or superficially addressed;
- By discussing the role of modern objective sleep tools (e.g., actigraphy, home-based monitoring) in both stroke risk prediction and post-stroke longitudinal sleep assessment;
- By providing a more cohesive synthesis and critical discussion of limitations, gaps, and future directions.

Thus, our review does not merely repeat prior overviews but aims to add an updated, stroke-centric, bidirectional, and methodology-aware perspective.

The medical literature points to an association between insomnia/poor sleep and stroke risk, but causality is not established. This is due to heterogeneity in definitions, confounding (especially undiagnosed obstructive sleep apnea [OSA]), reverse causality, and a dearth of prospective data with objective measures. In many studies, insomnia is self-reported or coded (ICD), rather than being objectively characterized, which complicates comparability across studies. The co-occurrence of OSA (a strong stroke risk factor itself) further clouds interpretation. This review synthesizes (1) mechanistic pathways linking insomnia and cerebrovascular risk, (2) observational evidence over the past decade, with evaluation of methodological strengths and biases, and (3) implications for clinical practice and research.

Notably, patients who survived stroke continue to demonstrate inter-limb transfer affects and practice-dependent motor learning, with better performance when training starts with the unaffected hemibody. Anxiety and attentional skills were strongly linked to motor function, emphasizing their importance in stroke recovery [18].

## 2. Methods

This work is a narrative review synthesizing the recent literature on chronic insomnia and stroke. It does not follow a registered systematic review protocol (e.g., PROSPERO), and inclusion was based on relevance, recency, and contribution to the field. A comprehensive, non-systematic literature search was conducted using MEDLINE (via PubMed), EMBASE, PsycINFO, CINAHL, and Web of Science/Scopus to identify relevant studies focusing on chronic insomnia and its association with stroke risk. Keywords used for research: “Stroke”, “cerebral infarction”, “cerebrovascular accident”, “brain infarct”, “cerebral hemorrhage”, “cerebrovascular event”, “cerebrovascular disease”, “insomnia”, “chronic insomnia”, “sleep disturbance”, and “sleep disorder”. Also, to improve and refine search results, truncations and Boolean operators (AND, OR) were used. To find recent evidence, the search focused on English-language publications from 2015 to 2025. To identify more relevant research, references from the selected studies were manually screened.

## 3. Pathophysiological Issues

**Figure 1:**
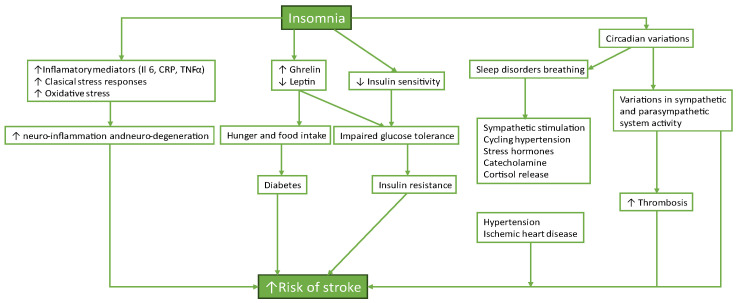
Insomnia and cardio-cerebral and metabolic dysfunction.

Several plausible biological pathways connect insomnia with cerebrovascular events—see Figure 1.

Both direct and indirect pathways that raise the risk factors for cardiovascular diseases are among the many effects of chronic insomnia on cardiovascular health [19]. The hypothalamic-pituitary-adrenal (HPA) axis can be triggered by prolonged stress and sleep deprivation, which raises cortisol and other stress hormone levels [20,21]. The condition’s enduring existence determines the appearance of systemic inflammation, sympathetic overactivity, and metabolic dysregulation, which all play an essential role in the pathogenesis of cardiovascular diseases. So, insomnia is linked to an increased risk for high blood pressure, coronary disease, underscoring the relevance of sleep health in preventing cerebrovascular disease [22].

### 3.1. Hypothalamic–Pituitary–Adrenal Axis Dysregulation

Chronic insomnia increases the secretion of corticotropin-releasing hormone (CRH), which leads to higher levels of cortisol (a stress hormone). Chronic activation of cortisol can lead to high blood pressure and inflammation, well-known risk factors for cardiovascular diseases. Prolonged cortisol release also affects glucose metabolism and lipid levels, potentially increasing the risk of cardiovascular disease [21,22,23].

Chronic insomnia may result in sustained activation of the CRH-ACTH-cortisol pathways, which can lead to hypertension, insulin resistance, and endothelial damage [23]. Some experimental sleep restriction studies (3–4 h nights) show lower morning cortisol and higher evening cortisol, indicating dysregulation under acute stress, but extrapolation to chronic insomnia is cautious [24,25,26].

### 3.2. Sympathetic Nervous System (SNS) Overactivity

Chronic insomnia patients have increased SNS activity, which causes elevated heart rates and blood pressure even while sleeping. This sympathetic dominance impairs the body’s natural parasympathetic recovery mechanisms and causes arterial rigidity, endothelial dysfunction, and increased peripheral resistance, all of which are risk factors for cardiovascular disease [27,28,29,30]. Sympathetic overactivity has also been linked to arrhythmogenesis, emphasizing the arrhythmic risk associated with sleeplessness and its potential contribution to sudden cardiac arrest [31].

Several studies found a link between sleep deprivation and both increased and decreased heart rate variability, implying a decrease in cardiac parasympathetic and/or a rise in sympathetic tone. A cross-sectional research of 30 young guys during university final examinations found that sleep deprivation, defined as sleep duration less than 80% of baseline across 4 weeks, was related to higher plasma norepinephrine levels (315 to 410 pg/mL, *p*  <  0.05) [32]. Autonomic dysregulation perpetuates sleep problems such as insomnia and fragmented sleep, as well as obesity, insulin resistance, and, eventually, an increased risk of coronary artery disease [33].

Individuals with chronic insomnia frequently have lower heart rate variability (HRV), which is a measure of autonomic nervous system function, indicating an imbalance between sympathetic and parasympathetic processes [34]. Reduced HRV has been linked to higher cardiovascular risk, indicating impaired cardiac autonomic regulation and an increased risk of arrhythmias [35]. Diminished HRV is a recognized risk marker in vascular disease and may be relevant to stroke [36].

### 3.3. Inflammatory Pathways

Chronic insomnia can lead to low-grade systemic inflammation, including high CRP, IL-6, and TNF-α levels. These pro-inflammatory cytokines are intimately linked to the development and progression of atherosclerosis, a major risk factor for cardiovascular disease [37]. Also, insomnia-related inflammation triggers endothelial dysfunction, an aspect of cardiovascular condition that predisposes individuals to thrombosis and plaque formation, increasing the risk of acute coronary artery disease [38,39].

Epidemiological data have also revealed a link between insomnia and high CRP levels. To this purpose, Parthasarathy et al. found a relationship between chronic persistent sleeplessness and cardiovascular mortality associated with elevated CRP levels [38]. Additionally, a major epidemiologic investigation (*N* = 4011) found a link between moderate to severe sleeplessness and higher CRP levels in men.

The interplay between inflammation and endothelial injury may be more directly relevant for cerebral vessels (microvascular damage, small vessel disease) than general coronary disease.

### 3.4. Oxidative Stress, Nitric Oxide (NO) Bioavailability, and Endothelial Homeostasis

Sleep is an essential immune modulator, therefore sleep deprivation may contribute to endothelial dysfunction by increasing vascular inflammation and reactive oxygen species (ROS) production [39,40]. Inflammation caused by acute or chronic sleep deprivation may occur from a chronically heightened sympathetic stress response and increased blood pressure [32,41,42]. While blood pressure normally drops 10–20% during nighttime sleep, the absence of dipping or elevations during sleep deprivation activates the endothelium, causing the release of inflammatory mediators, vascular adhesion molecules, and coagulation factors that contribute to endothelial dysfunction [43]. This would also explain why persistent sleep restriction, which prevents the resolution of this inflammatory response, is associated with chronic low-grade inflammation and inflammatory diseases.

Acute inflammation affects endothelial function by increasing oxidative stress in the vasculature and decreasing NO bioavailability [43]. ROS damages endothelial cells, causes apoptosis, and promotes cell proliferation, decreasing NO bioavailability directly or indirectly. Superoxide anions (O^2−^) rapidly react with NO to produce peroxynitrite (ONOO^−^), which can damage lipids, proteins, and DNA. This response decreases NO bioavailability while increasing ROS within the endothelium. ROS additionally reduce NO bioavailability by promoting endothelial nitric oxide synthase (eNOS) uncoupling, producing ROS rather than NO. NO bioavailability is reduced, which compromises endothelial-dependent vasodilation [41,42,43].

ET-1, when attached to endothelial ETA receptors, causes vascular smooth muscle constriction, which opposes NO’s activities. ETA receptor binding also promotes ROS production and inhibits NO synthesis and activities, implicating ET-1 in the etiology of endothelial dysfunction [44,45,46]. One study in our evaluation discovered that short sleepers had higher ET-1-mediated vasoconstrictor tone [45]. Acute sleep deprivation may affect the NO/ET-1 balance. According to one study, acute TSD raised plasma ET-1 levels while leaving plasma nitrite levels unchanged, favoring vasoconstriction and endothelial dysfunction [47,48,49]. As a result, low-grade inflammation, elevated ROS, and their effects on the vasodilator/vasoconstrictor balance are likely key contributors to endothelial dysfunction caused by acute and chronic sleep loss.

### 3.5. Circadian Dysregulation/Sleep–Wake Pattern Irregularity

Irregular sleep timing (irregular bedtimes or wake-up times) may impair circadian control of blood pressure, glucose metabolism, and vascular repair. Indeed, a recent UK Biobank study using actigraphy-derived sleep regularity found that greater day-to-day variability in sleep schedule was associated with 26% higher risk of stroke and major cardiovascular events, independent of sleep duration [50]. This suggests that regularity may matter as much as the total amount of sleep.

Although insomnia may be linked to cerebrovascular events, there is a lack of prospective mediation models (insomnia → mediator → stroke), and the specificity of effects on brain vasculature vs. systemic arteries is unknown.

### 3.6. Metabolic Effects

**Figure 2:**
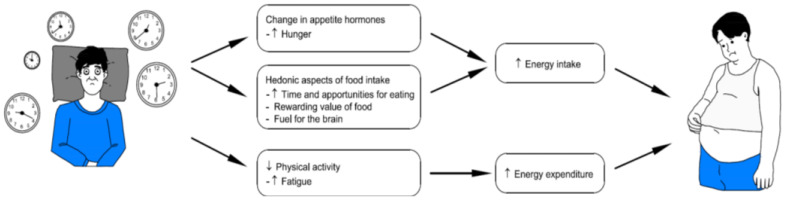
Relationship between sleep and metabolic syndrome.

Individuals with chronic insomnia are more likely to have metabolic abnormalities such as insulin resistance obesity, and dyslipidemia, all established stroke risk factors [51]. Insomnia has been confirmed to interfere with ghrelin and leptin levels—hormones that regulate hunger and satiety—resulting in increased appetite, weight gain, and a higher incidence of obesity, which is an important risk factor for cardiovascular disease [16,43,52,53,54]. In addition, insulin resistance, which is typically seen in people with poor sleep quality, increases the likelihood of developing type 2 diabetes, another main cardiovascular risk factor [51,55]—(see Figure 2).

Data from the UK Biobank and the Meta-analyses of Glucose and Insulin-Related Characteristics Consortium (MAGIC) were evaluated to assess the impact of five patient-reported sleep characteristics: insomnia, sleep duration, daytime sleepiness, napping, and chronotype on HbA1c [56]. A higher frequency of sleeplessness (usually vs. occasionally or rarely/never) was observed to be related with higher HbA1c. The results remained significant, although the point estimates were somewhat reduced after removing participants with diabetes. This study suggests that insomnia raises HbA1c levels. These findings could have significant implications for creating and evaluating sleep-improvement methods to lower hyperglycemia and prevent diabetes [56,57].

Sleeping for less than 6 h was linked to obesity, with higher evidence for women than males [58,59]. Similarly, short sleepers had an increased fat mass index and were 1.22 times more likely to have general (OR 1.22, 95% CI 1.03–1.45) and abdominal (OR 1.32, 95% CI 1.10–1.58) obesity [60,61,62,63,64].

The association between long or short sleep duration and the risk of stroke has been extensively studied. Short sleep duration or sleep deprivation can increase the risk of stroke due to the impact on the cardiovascular system and metabolism, leading to higher sympathetic activity, increased cortisol secretion, and inflammation [3,56,65]. While these mechanisms provide biological plausibility, causal pathways specifically linking insomnia to stroke are not yet firmly established, as mediation studies remain sparse.

## 4. Epidemiological Evidence: From Insomnia to Stroke

### 4.1. Insomnia as a Predictor for Stroke

Over the past ten years, an increasing number of observational studies have looked at the relationship between incident stroke and chronic insomnia, consistently indicating a slightly increased risk. With an odds ratio of approximately 1.3 to 1.5, people with chronic hypertension, were more likely to experience symptoms of insomnia, according to McCarthy et al. in 2023 [3]. In accordance with a study published in 2003, persistent insomnia paths showed a significant correlation with a hazard ratio of 1.25 over six years, although transient insomnia did not [66]. Another study found a hazard ratio of 1.42 between the UK Biobank cohort’s highest and lowest quartiles of sleep regularity, indicating that irregular sleep patterns may increase risk even more [50]. Similar findings, were reported in cohort studies carried out in Taiwan: Huang and colleagues found a hazard ratio of 1.35 for ICD-coded insomnia after propensity-score matching, while Wu and colleagues reported a hazard ratio of 1.4, particularly among older adults [67,68]. Another study publish in 2020 found a hazard ratio of 1.2 using short sleep duration (less than 6 h) as a stand-in for insomnia; however, assessment of chronicity was limited by a single baseline measurement [69]. Lastly, despite residual confounding being a concern, Ao and colleagues reported a hazard ratio of 1.3 in patients with post-traumatic brain injury [70]. All of these studies point out that is a slightly elevated risk of stroke for chronic insomnia, particularly when it is persistent or accompanied by irregular sleep patterns (see Table 1).

Studies encountered confounding concerns, frequently overlooking characteristics such as obstructive sleep apnea (OSA), physical activity, food, and socioeconomic status, particularly in administrative datasets. While the participant cohorts were largely representative, clinical subgroups limited generalizability. Misclassification of insomnia was widespread due to dependence on self-reports, while accelerometry gave more objective data. There were no interventions; thus, deviations were not an issue. Dropout was low, and administrative datasets minimized missing data. Stroke outcomes were typically accurately recorded; however, self-reports could contribute to misclassification. Overall, the majority of studies exhibited a moderate risk of bias, with administrative cohorts and case–control designs being more susceptible.

A systematic review (4 studies) published in 2022 found that three of four reported positive associations between chronic insomnia disorder and stroke, but highlighted heterogeneity in definitions and measurement [11].

In a cohort and case–control study it was indicated that insomnia/poor sleep quality are associated with increased risk of cerebrovascular disease, but note the complexity of effect modification and confounding [7].

In some large administrative cohorts (e.g., in Taiwan), ICD-coded insomnia was associated with modestly elevated hazard ratios for ischemic stroke (e.g., HR ~1.35 after propensity matching) [37].

Trajectory analyses (e.g., persistent insomnia vs. transient) suggest that persistent insomnia or worsening sleep irregularity confer higher risk than transient symptoms.

However, most studies do not robustly adjust for OSA, nor do they always validate insomnia exposure multiple times. Residual confounding (physical activity, diet, socioeconomic status, mental health) remains a substantial threat. The magnitude of associations is modest (HRs ~1.2–1.4 in many studies) and inconsistent across populations; so, any causal claim should remain tentative [71,72,73].

Given the heterogeneity of designs, populations, definitions, and confounder adjustment, a meta-analysis restricted to insomnia-to-stroke studies is challenging. Until that is feasible, statements such as “modest increase in risk” should be taken as hypothesis-generating, not definitive.

### 4.2. Sleep Duration, Sleep Regularity, and Stroke Risk

Sleep duration (both short and long) and sleep–timing irregularity have been extensively investigated as predictors of stroke. A consistent finding across prospective studies and pooled analyses is a non-linear (U- or J-shaped) relationship between habitual sleep duration and stroke risk, with the lowest risk typically observed at about 7 h per night and progressively higher risk at both shorter and longer durations.

Early large prospective work and pooled analyses found that long sleep shows the strongest and most consistent association with stroke. According from a study that combined data from the EPIC-Norfolk cohort with an updated meta-analysis and reported pooled hazard ratios of ~1.45 (95% CI 1.30–1.62) for long sleep (commonly > 8 h) and ~1.15 (95% CI 1.07–1.24) for short sleep (commonly < 6 h), indicating a stronger signal for extended sleep time in relation to future stroke [74].

Subsequent dose–response meta-analyses confirmed the non-linear relation.Some authors used data from multiple prospective cohorts (hundreds of thousands of participants) and demonstrated a J-shaped association between sleep duration and total stroke risk, with the nadir around 7 h. On a dose scale, each hour of sleep beyond ~7 h was associated with progressively higher stroke risk, while reductions below 7 h produced smaller incremental hazard increases [75].

A 2022/2023 wave of pooled analyses and reviews reinforced these patterns and highlighted heterogeneity across studies. For example, a study published in 2022 reported that both short and long sleep durations were associated with increased stroke incidence and mortality, but again, the effect for long sleep tended to be larger and more robust across sensitivity analyses [76].

Important caveats apply. Many of the cohort studies and meta-analyses rely on self-reported sleep duration at baseline (single timepoint), which risks misclassification and fails to capture chronicity or changes over time. Several authors therefore emphasize that long sleep may be a marker of subclinical disease, frailty, low activity, depression, or other conditions that themselves increase stroke risk rather than a direct causal exposure. These alternative explanations are plausible and may account for some of the stronger association seen with long sleep [74,76].

Beyond average duration, sleep regularity and timing have emerged as potentially important, independent predictors. Large device-measured (actigraphy) studies in UK Biobank participants show that irregular sleep timing or low sleep regularity indices are associated with higher risk of adverse outcomes (including cardiovascular events and mortality), and recent device-based analyses suggest regularity may add predictive information beyond duration alone. For example, actigraphy-based analyses from UK Biobank cohorts have linked lower sleep regularity with higher cardiovascular risk indices and all-cause and cardiovascular disease (CVD) mortality. A recent prospective device-based study reported that poorer sleep regularity predicted major adverse cardiovascular events independently of sleep duration. These findings imply that variability in sleep timing (fragmentation/irregular schedules) could contribute to stroke risk through circadian disruption, autonomic instability, and metabolic dysregulation [50,77,78].

Finally, a number of cohort studies explored stroke subtypes. Some evidence suggests short sleep may be more consistently associated with ischemic stroke, whereas long sleep has sometimes shown stronger associations with hemorrhagic stroke or with overall stroke mortality, but these subtype results are inconsistent across populations and likely underpowered. For example, population studies in East Asia and Europe have reported varying subtype patterns (some reporting long sleep associated with hemorrhagic stroke in women; others reporting short sleep associated with ischemic events), emphasizing that conclusions about subtype-specific risk remain tentative [69,79].

In summary, the best current evidence supports a U-shaped association between habitual sleep duration and stroke risk (nadir ≈ 7 h), with long sleep showing the larger and more consistent association. However, heterogeneity in exposure assessment (self-report vs. device), confounding (comorbidity, depressive symptoms, low activity), the possibility of reverse causation (long sleep as a marker of subclinical disease), and inconsistent subtype results mean that causal interpretation must be cautious. Newer device-based measures of sleep regularity strengthen the case that dimensions of sleep beyond average duration (timing and variability) are relevant to cerebrovascular risk and deserving of inclusion in future prospective and mechanistic studies. Thus, sleep duration and regularity may be complementary exposure dimensions to insomnia per se; however, mechanistic and longitudinal specificity for stroke outcomes remains limited.

### 4.3. Pooled Analyses and Risk Patterns

Evidence from pooled cohort studies highlights the complexity of the relationship between sleep duration, insomnia, and vascular risk. Large-scale analyses suggest that insomnia and short sleep are consistently associated with adverse cardiovascular outcomes, although the strength of association varies across studies. For example, a recent meta-analysis including more than 1 million participants demonstrated that individuals reporting chronic insomnia or very short sleep (<5 h per night) had a 69% higher risk of myocardial infarction; similar but somewhat attenuated associations were observed for stroke [64]. Importantly, these findings remained significant even after adjustment for traditional vascular risk factors such as hypertension, diabetes, and dyslipidemia, suggesting that sleep disruption may act as an independent contributor to vascular pathology.

Beyond dichotomous definitions of short versus long sleep, dose–response analyses provide additional insights. Several meta-analyses have documented a U-shaped relationship, with both curtailed and prolonged sleep associated with increased vascular risk [69,80]. Specifically, each one-hour reduction below seven hours of sleep has been linked to an approximately 5% increased risk of stroke, while each one-hour increment above seven hours confers an even greater relative risk, up to 18% in some cohorts. This non-linear association suggests distinct underlying mechanisms: short sleep may exacerbate sympathetic activation, inflammation, and metabolic dysregulation, whereas long sleep could reflect underlying comorbidity, reduced physical activity, or subclinical neurodegenerative processes.

### 4.4. Confounding and Uncertainty

OSA remains a major unmeasured confounder in many studies. Reverse causality (prodromal cerebrovascular disease impairing sleep) cannot be excluded, particularly in older cohorts. Thus, causality remains uncertain and associations should be interpreted cautiously [7].

## 5. Bidirectional Links: Stroke Impact on Sleep

Given a bidirectional model, it is crucial to evaluate how stroke influences insomnia/sleep disturbances, and how those in turn relate to recovery, function, and prognosis (see Table 2).

### 5.1. Epidemiology of Post-Stroke Sleep Disturbances

Sleep disorders are highly prevalent following stroke, affecting between 30% and 70% of survivors depending on the setting, diagnostic approach, and time since the event [89,90]. Recent hospital-based data confirm this high burden: A report from 2025 showed that over half of stroke patients in a Saudi tertiary care center exhibited clinically significant sleep disturbances, most commonly insomnia and obstructive sleep apnea (OSA), with a strong association between poor sleep and stroke recurrence [81]. These findings are consistent with earlier European and Asian cohorts, highlighting that sleep disorders represent both a risk factor for stroke occurrence and a common consequence of cerebrovascular injury [86,89,90].

Objective and subjective assessments further underscore this burden. Fleming et al. (2021) showed that even in the chronic phase of stroke, patients with incomplete motor recovery frequently reported sleep inefficiency and irregular sleep patterns, corroborated by actigraphy [82]. More recently, a 2025 study) used polysomnography and found that patient- and caregiver-reported sleep problems correlated with measurable abnormalities in sleep architecture, particularly reduced REM sleep and increased arousal indices [83]. Together, these data suggest that sleep disorders remain underdiagnosed yet highly prevalent in post-stroke populations worldwide.

### 5.2. Functional and Cognitive Consequences

The clinical consequences of post-stroke sleep disturbances are increasingly recognized as substantial. Poor sleep not only compromises rehabilitation but also predicts long-term cognitive and functional decline. 2025 large Norwegian cohort, s demonstrated that both insufficient (<6 h) and excessive (>9 h) sleep durations were associated with worse global cognition at two years, reinforcing the U-shaped risk relationship [84]. Similarly, another study published in 2020 found that poor sleep quality was an independent predictor of reduced functional independence during rehabilitation, even after adjusting for age, stroke severity, and comorbidities [85].

Cognitive sequelae have been highlighted in population-level studies. Trajectory analysis in over 14 international cohorts, showed that cognitive decline often accelerates after stroke, and comorbid sleep disturbances may exacerbate this trajectory [87]. Complementary evidence published in 2024 ) indicated that insomnia symptoms in stroke survivors were linked not only to poorer quality of life but also to an increased risk of all-cause mortality, underscoring prognostic relevance [35].

The interplay between depression, cognition, and sleep adds complexity. A scoping review emphasized that sleep disturbances frequently co-occur with mood disorders and cognitive impairment after stroke, creating a synergistic negative effect on recovery and outcomes [88].

Collectively, these findings underscore that post-stroke sleep disturbances are not benign. They are epidemiologically common, mechanistically diverse, and strongly linked to functional impairment, cognitive decline, and mortality. Systematic incorporation of sleep evaluation into stroke care pathways—through actigraphy, polysomnography, or validated questionnaires—could facilitate early detection and targeted intervention, potentially improving long-term outcomes.

### 5.3. Overlap with Poststroke Breathing Disorders

Sleep-disordered breathing (SDB), especially obstructive sleep apnea (OSA), is highly prevalent poststroke (estimates 65% or more) [91,92,93].

Meta-analytic data suggest modest but directionally positive associations between OSA and worse poststroke complications (cognitive impairment, insomnia, fatigue, recurrence), especially in patients with more severe strokes [94].

Because insomnia and OSA often co-occur or interact, teasing out the independent contributions of insomnia vs. SDB in poststroke trajectories is an ongoing challenge. The overlapping pathophysiology (intermittent hypoxia, arousals, sympathetic activation) complicates attribution.

### 5.4. Advances and Challenges in Objective Sleep Measurement

A major limitation across epidemiological and poststroke sleep literature has been reliance on self-report or single-night PSG. Modern tools offer opportunities to improve exposure and outcome measurement:

- Actigraphy/wearables: Noninvasive, continuous measurement of rest–activity patterns, sleep duration, fragmentation, and regularity. Widely used in geriatrics and sleep epidemiology. Their integration into stroke research is limited but growing [95,96].
- Ambulatory/Home Sleep Apnea Testing (HSAT): Portable monitors (e.g., ApneaLink) have been deployed post-TIA or stroke, achieving usable data in ~80% of patients and detecting high OSA prevalence [95,96].
- Home-based polysomnography/portable EEG: New-generation portable PSG systems may allow for a multi-night, in-home sleep architecture assessment, though validation in stroke populations is ongoing [95,97].
- Sleep regularity metrics/AI analytics: Metrics such as Sleep Regularity Index, intra-individual variability, and machine learning–derived patterns may capture fragmentation and circadian disruption better than average sleep time [95].
- Integrated multimodal monitoring: Combining sleep sensors with blood pressure, heart rate variability, actimetry, and movement sensors may allow for real-time cerebrovascular risk profiling [95].

However, challenges remain: device validation in stroke populations, adherence, data processing, cost, and regulatory acceptance. Moreover, translating device metrics into clinical risk models (for stroke incidence or recovery) is in early stages.

## 6. Discussions

### 6.1. Summary of Evidence and Interpretation

**Figure 3:**
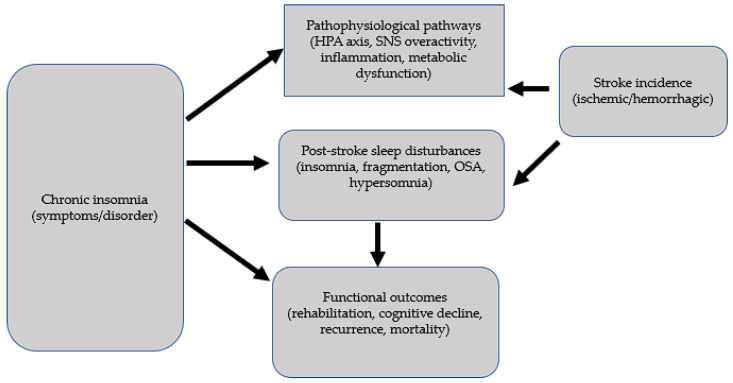
Insomnia-Stroke: a bidirectional issue.

- Observational studies suggest that chronic insomnia or persistent sleep disturbance are modestly associated with increased risk of incident stroke, though causality remains speculative.
- Meta-analytic evidence for sleep duration indicates a U-shaped relationship with stroke, reinforcing the idea that both insufficient and excessive sleep durations carry risk [82,91].
- Stroke survivors frequently develop insomnia or sleep disturbance (pooled prevalence 50%) and sleep–architecture metrics poststroke correlate with functional and cognitive outcomes [18,96,97,98,99].
- The bidirectionality (insomnia → stroke and stroke → insomnia) complicates causal inferences and necessitates approaches that disentangle temporal relationships (see Figure 3) [100,101].
- Objective monitoring tools hold promise for improving exposure and outcome measurement, reducing misclassification, and enabling dynamic risk assessment.

### 6.2. Limitations and Methodological Challenges

- Heterogeneity in insomnia definitions: different questionnaires, thresholds, and exposure windows impede comparability.
- Confounding and OSA: many studies lack objective OSA assessment; residual confounding is likely substantial.
- Reverse causality risk: subclinical cerebrovascular disease or brain aging may cause sleep disturbance before clinical stroke is diagnosed.
- Sparse longitudinal and repeated-measures designs: many studies rely on baseline sleep measurement alone, neglecting chronicity dynamics.
- Lack of randomized or interventional trials: no large trials have definitively tested whether insomnia treatment reduces stroke risk.
- Stroke subtype, severity, lesion heterogeneity: differential vulnerability of brain regions to insomnia effects is underexplored.
- Measurement error: self-report sleep measures are prone to recall bias; single-night PSG may not reflect habitual sleep.

Given these constraints, statements that “insomnia increases stroke risk independently of vascular risk factors” should remain tempered.

### 6.3. Clinical Implications

- Even in the absence of causal certainty, insomnia may function as a clinical risk marker in populations already at vascular risk. Screening (e.g., ISI, PSQI) may help stratify patients for further evaluation or prevention.
- In stroke patients, routine poststroke sleep assessment (e.g., Sleep Condition Indicator) is feasible and valid (SCI validated in stroke populations) [100].
- Given high prevalence of OSA in stroke survivors, concurrent screening/treatment is prudent.
- Behavioral insomnia therapies (e.g., CBT-I) may offer potential for modifying risk or improving recovery, though large trials are lacking.
- Sleep monitoring (actigraphy, wearables) might eventually inform personalized rehabilitation timing, fatigue management, and recurrence risk stratification.

### 6.4. Recommendations for Future Research

- Large prospective cohorts with repeated, multimodal sleep assessments (self-report + actigraphy/portable PSG) and long follow-up.
- Mediation modeling: concurrent biomarker collection (inflammation, autonomic indices) to test mechanistic pathways.
- OSA integration: objective measurement and adjustment for OSA should be mandatory in future insomnia–stroke studies.
- Randomized intervention trials: e.g., CBT-I, digital sleep therapy vs. controls, with stroke or surrogate vascular outcomes (e.g., white-matter hyperintensity progression, carotid intima-media thickness).
- Use of AI/machine learning: to derive high-dimensional sleep phenotypes, cluster insomnia subtypes, and predict stroke risk.
- Subgroup and effect modification analyses: sex, age, lesion location, stroke subtype (ischemic vs. hemorrhagic).
- Implementation science: integrating wearable sleep monitoring into stroke clinics, testing adherence, feedback loops, and cost-effectiveness.

## 7. Conclusions

The existing body of observational evidence supports an association between chronic insomnia/persistent sleep disturbance and a modest increase in incident stroke risk, though methodological limitations preclude definitive causal claims. Conversely, insomnia and other sleep disturbances are common after stroke and correlate with poorer functional recovery, cognition, and survival. Emerging objective monitoring technologies (actigraphy, portable PSG, AI analytics) offer promise for refining both risk prediction and poststroke trajectories. To move from association to causation (and eventually intervention), future research must adopt rigorous designs: standardized definitions, repeated measures, objective methods, OSA adjustment, and interventional frameworks. Ultimately, targeting insomnia may evolve from a risk marker to a modifiable component of cerebrovascular prevention and rehabilitation.

## Figures and Tables

**Table 1:** Studies and Reviews Examining Insomnia/Sleep Disturbance and Stroke Risk.

Author/Year	Design & Population	Sleep Exposure/Assessment	Prevalence/Findings	Functional/Clinical Impact	Notes/Limitations
McCarthy et al., 2023 [3]	INTERSTROKE case–control (>4500 stroke vs. controls)	Self-reported sleep duration, insomnia symptoms, OSA features	Short and long sleep, poor quality, and snoring linked to higher stroke risk	Identified OSA and irregular sleep as independent stroke predictors	Recall bias; global population heterogeneity
Matas et al., 2024 [7]	Narrative review	Literature synthesis	Summarized mechanistic and epidemiologic evidence	Emphasized insomnia as modifiable stroke risk factor	Not systematic; no quantitative synthesis
Silva et al., 2022 [11]	Systematic review (observational studies)	Clinical insomnia disorder (DSM/ICD)	Chronic insomnia associated with increased stroke risk	Independent vascular risk suggested	Few studies; heterogeneity of definitions
Chaput et al., 2025 [50]	UK Biobank (*n* = 72,269); prospective	Actigraphy-derived sleep regularity	Irregularity predicted higher MACE (incl. stroke)	Device-based monitoring promising for risk prediction	Stroke-specific outcomes less granular
Sawadogo & Adera, 2023 [66]	Cohort (Neurology abstract)	Insomnia symptom trajectories	Persistent insomnia linked to higher stroke incidence	Longitudinal assessment valuable	Limited details; abstract-only report
Huang et al., 2024 [67]	Kailuan cohort, >50,000 adults	Composite “healthy sleep pattern”	Healthy sleep linked to lower stroke risk	Multidomain sleep index may aid prevention	Asian population; external validation needed
Titova et al., 2020 [69]	Swedish cohorts (*n* = 40,000+) + Mendelian randomization	Questionnaire + genetic data	Long sleep (≥9 h) HR ~1.44 for ischemic stroke	Supports possible causal link	Generalizability limited; MR assumptions
Ao et al., 2017 [70]	Taiwan national cohort (TBI patients with insomnia)	Insurance data (ICD codes)	Early insomnia post-TBI ↑ risk of stroke	Suggests post-injury insomnia as enhancer	No objective sleep data

**Table 2:** Post-Stroke Sleep Disturbances: Epidemiology and Consequences.

Author/Year	Design & Population	Sleep Exposure/Assessment	Prevalence/Findings	Functional/Clinical Impact	Notes/Limitations
Alabdali et al., 2025 [81]	Retrospective, hospital-based; stroke patients in Saudi Arabia	Medical records, questionnaires	>50% of patients had sleep disorders (insomnia, OSA most common)	Linked to higher recurrence risk	Retrospective, single-center, may under-detect sleep disorders
Fleming et al., 2021 [82]	Cross-sectional; chronic stroke survivors with incomplete motor recovery	Self-reported sleep measures + actigraphy	High prevalence of poor sleep quality, irregular patterns	Sleep inefficiency is associated with worse motor outcomes	Small sample, chronic stage only
Tayade et al., 2025 [83]	Cross-sectional, post-stroke patients with sleep complaints	Polysomnography + caregiver/patient report	Objective abnormalities: reduced REM, high arousals	Confirmed subjective complaints; supports link to disrupted recovery	Single-center, limited follow-up
Ihle-Hansen et al., 2025 [84]	Prospective cohort; Norwegian Cognitive Impairment After Stroke study	Self-reported sleep duration	Both short (<6 h) and long (>9 h) sleep are linked to poorer cognition at 2 years	U-shaped relationship; worsened long-term cognitive outcomes	Observational, self-report bias
Iddagoda et al., 2020 [85]	Prospective cohort; 112 stroke rehabilitation patients	Pittsburgh Sleep Quality Index (PSQI)	65% poor sleep quality	Poor sleep predicted reduced independence post-rehab	Small cohort, limited generalizability
Curci et al., 2018 [86]	Cross-sectional; chronic stroke outpatients	Structured questionnaires	High prevalence of chronic insomnia and fragmented sleep	Associated with lower functional recovery scores	Small, cross-sectional
Sawadogo et al., 2024 [66]	Prospective cohort; stroke survivors	Self-reported insomnia symptoms	Insomnia linked to higher all-cause mortality	Highlights the prognostic role of sleep after stroke	Self-report, residual confounding
Lo et al., 2024 [87]	Pooled analysis of 14 cohorts; >20,000 stroke survivors	Neuropsychological testing + stroke records	Accelerated cognitive decline after stroke	Sleep disturbance may exacerbate the decline trajectory	Not sleep-focused, indirect evidence
Chan, 2024 [88]	Scoping review	Literature synthesis	Frequent co-occurrence of sleep disturbance, depression, and cognitive impairment	Describes synergistic negative effects	Narrative, not quantitative
Bassetti et al., 2020 [89]	Guideline/consensus statement	Evidence synthesis	Sleep disorders are common post-stroke	Emphasizes the clinical importance of screening and treatment	Consensus-based, not new data

## Data Availability

Not applicable.
